# Premotor cortices differentially influence contralateral motor cortex

**DOI:** 10.1093/cercor/bhag163

**Published:** 2026-10-12

**Authors:** Elnaz Allahverdloo, Larissa K Chiu, Amanda O’Farrell, Nesrine Harroum, Numa Dancause, Jason L Neva

**Affiliations:** Centre de recherche de l’institut universitaire de gériatrie de Montréal (CRIUGM), 4545 chemin Queen-Mary, Montréal, Québec H3W 1W4, Canada; Département de neurosciences, Faculté de médecine, Université de Montréal, Pavillon Paul-G.-Desmarais, 2960, chemin de la Tour, Montréal, Québec H3T 1J4, Canada; Centre Interdisciplinaire de Recherche sur le Cerveau et l’Apprentissage (CIRCA), Pavillon 3744, rue Jean-Brillant, Université de Montréal, Montréal, Québec H3T 1P1, Canada; Centre de recherche de l’institut universitaire de gériatrie de Montréal (CRIUGM), 4545 chemin Queen-Mary, Montréal, Québec H3W 1W4, Canada; Centre de recherche de l’institut universitaire de gériatrie de Montréal (CRIUGM), 4545 chemin Queen-Mary, Montréal, Québec H3W 1W4, Canada; École de kinésiologie et des sciences de l'activité physique (EKSAP), Faculté de médecine, Université de Montréal, 2100 boulevard Édouard-Montpetit, Montréal, Québec H3C 3J7, Canada; Centre de recherche de l’institut universitaire de gériatrie de Montréal (CRIUGM), 4545 chemin Queen-Mary, Montréal, Québec H3W 1W4, Canada; École de kinésiologie et des sciences de l'activité physique (EKSAP), Faculté de médecine, Université de Montréal, 2100 boulevard Édouard-Montpetit, Montréal, Québec H3C 3J7, Canada; Département de neurosciences, Faculté de médecine, Université de Montréal, Pavillon Paul-G.-Desmarais, 2960, chemin de la Tour, Montréal, Québec H3T 1J4, Canada; Centre Interdisciplinaire de Recherche sur le Cerveau et l’Apprentissage (CIRCA), Pavillon 3744, rue Jean-Brillant, Université de Montréal, Montréal, Québec H3T 1P1, Canada; Institut Courtois d’innovation biomédicale (CI2B), Faculté de médecine, Université de Montréal, Pavillon Paul-G.-Desmarais, 2960 chemin de la Tour, Montréal, Québec H3T 1J4, Canada; Centre de recherche de l’institut universitaire de gériatrie de Montréal (CRIUGM), 4545 chemin Queen-Mary, Montréal, Québec H3W 1W4, Canada; Centre Interdisciplinaire de Recherche sur le Cerveau et l’Apprentissage (CIRCA), Pavillon 3744, rue Jean-Brillant, Université de Montréal, Montréal, Québec H3T 1P1, Canada; École de kinésiologie et des sciences de l'activité physique (EKSAP), Faculté de médecine, Université de Montréal, 2100 boulevard Édouard-Montpetit, Montréal, Québec H3C 3J7, Canada

**Keywords:** dorsal/ventral premotor cortex, interhemispheric inhibition, ipsilateral silent period, primary motor cortex, transcranial magnetic stimulation

## Abstract

Dorsal (PMd) and ventral (PMv) premotor cortices can modulate contralateral primary motor cortex (M1) excitability, but their distinct interhemispheric influence via transcranial magnetic stimulation (TMS) remains unclear. Single-pulse TMS over PMd, PMv and M1 assessed transcallosal inhibition via the ipsilateral silent period (iSP). Dual-site TMS examined short-(10 ms inter-stimulus interval [ISI]), long-(50 ms ISI) and non-callosal-(0 ms ISI) interhemispheric inhibition (IHI). An iSP was elicited from PMd, PMv, and M1, with distinctly evoked iSP parameters. The iSP magnitude was greatest from M1, followed by PMd and then PMv, while iSP duration was greatest for M1 and showed no differences between PMd and PMv. Dual-site TMS revealed that PMd and M1 inhibited contralateral M1 excitability across all ISIs, while PMv showed inhibition at 0- and 50-ms ISIs. PMd and M1 demonstrated greater short-IHI compared to PMv, all demonstrating similar long-IHI, and PMd demonstrating greater non-callosal-IHI than M1. PMv displayed distinct IHI across ISIs, PMd showed differences across most ISIs and M1 demonstrated the fewest differences across ISIs. Longer iSP duration related to greater long-IHI magnitude elicited from PMd and PMv. Our findings demonstrate differential IHI from PMd and PMv on contralateral M1, which may inform neuromodulation strategies in rehabilitation contexts.

## Introduction

Goal-directed movements and motor learning rely on a distributed network of motor-related regions, with the primary motor (M1) and premotor (PMC) cortices playing critical, yet distinct, roles (Rizzolatti and Luppino 2001; Hoshi and Tanji 2007; Van Malderen et al. 2023). While M1 is traditionally viewed as the main driver of movement execution and a principal source of corticospinal output, PMC—encompassing its dorsal (PMd) and ventral (PMv) subregions—contributes to motor planning, preparation, and execution, and has its own corticospinal output (Hoshi and Tanji 2004; Chouinard and Paus 2006; Denyer et al. 2023). Critically, PMC can shape M1 output via cortico-cortical connections within and across hemispheres, as well as through subcortical pathways (Bäumer et al. 2006). Understanding the precise nature of these PMC-M1 interactions is of particular importance, as they appear to be implicated in the recovery of motor function following stroke (Frost et al. 2003; Dancause et al. 2005; Paul et al. 2023). However, such reorganization is not uniformly beneficial, as altered PMC-M1 interactions have been linked to motor deficits following stroke (Grefkes et al. 2008; Bestmann et al. 2010), highlighting the importance of understanding how distinct premotor subregions influence contralateral M1.

PMC can exert modulatory influence on ipsilateral M1 output through direct cortico-cortical connections, refining movement parameters such as force and grip-type (Davare et al. 2008; Davare et al. 2009). Dual-site transcranial magnetic stimulation (TMS) studies have shown that PMd and PMv differentially modulate ipsilateral M1 excitability, with distinct patterns of inhibition and facilitation depending on the subregion stimulated and stimulus parameters (Davare et al. 2008; Groppa et al. 2012). These ipsilateral influences are supported by anatomical connectivity, with both PMd and PMv sending direct projections to ipsilateral M1 via short-range cortico-cortical fibers (Dancause 2006; Schulz et al. 2015; Dea et al. 2016). However, the influence of PMC on M1 extends beyond the ipsilateral hemisphere to encompass interhemispheric interactions, which is the focus of the present study.

Anatomical evidence from tracing studies in non-human primates shows that PMC has many transcallosal connections with contralateral M1 (Marconi et al. 2003; Boussaoud et al. 2005; Dancause et al. 2007). In humans, diffusion-based tractography confirms the transcallosal representation of PMC within the corpus callosum and identifies PMd as a key contributor to the interhemispheric fiber tracts in the cortical motor network (Zarei et al. 2006; Ruddy et al. 2017). Neurophysiological evidence from dual-site TMS further supports this transcallosal connectivity, demonstrating that PMd can modulate contralateral M1 excitability across distinct interhemispheric inhibitory windows (Ni et al. 2009). Structural evidence also demonstrates interhemispheric connectivity between PMv and contralateral M1, though these connections appear less prominent than those from PMd (Boussaoud et al. 2005; Dancause et al. 2007; Ruddy et al. 2017). This notion is supported by dual-site TMS studies demonstrating complex PMv-to-M1 interhemispheric modulations (Fiori et al. 2017). Despite gathering anatomical and neurophysiological evidence for PMd-to-M1 and PMv-to-M1 interhemispheric connectivity, the precise nature of these influences—particularly how PMd and PMv differentially modulate contralateral M1 excitability—remains incompletely understood.

TMS offers complementary methods to probe the neurophysiology of interhemispheric inhibition and facilitation (Ferbert et al. 1992; Chen et al. 2008). Single-pulse TMS can elicit the ipsilateral silent period (iSP), a transient suppression of voluntary muscle activity in the contracting limb ipsilateral to the stimulated hemisphere, which reflects transcallosal inhibition (Ferbert et al. 1992; Hupfeld et al. 2020). The iSP provides a broad index of transcallosal inhibitory connectivity—including the silent period magnitude, onset, and duration—and is understood to be driven primarily by cortical mechanisms via the corpus callosum, though precise mediating mechanisms remain an area of investigation (Meyer et al. 1995; Boroojerdi et al. 1996; Hupfeld et al. 2020). Dual-site TMS offers a complementary approach that can further probe mechanisms of interhemispheric inhibition (IHI) and facilitation (IHF) (Ferbert et al. 1992; Chen et al. 2008; Neva et al. 2020). Dual-site TMS employs a conditioning stimulus (CS) over one hemisphere followed by a test stimulus (TS) over contralateral M1, allowing the examination of IHI/IHF across distinct temporal windows (Ferbert et al. 1992; Chen et al. 2008; Van Malderen et al. 2023). Two distinct temporal windows of M1-to-M1 IHI have been demonstrated, including short-IHI (inter-stimulus intervals [ISIs] of 8 to 12 ms) and long-IHI (ISIs of 30 to 50 ms; (Ni et al. 2009)). Each of these IHI phases reflects distinct underlying mechanisms of inhibition toward the contralateral M1, with evidence that long-IHI is mediated by postsynaptic gamma-aminobutyric acid type B (GABA_B_) receptors, and short-IHI being less understood (Irlbacher et al. 2007). Importantly, long-IHI elicited from M1 has been shown to correlate with iSP duration, suggesting that these two measures capture overlapping transcallosal mechanisms of influence on contralateral M1 (Chen et al. 2003).

To date, the iSP has been employed exclusively to study M1-to-M1 interhemispheric communication, and its potential to assess transcallosal inhibition originating from non-M1 regions such as PMd and PMv has not been investigated (Neva et al. 2020). Dual-site TMS studies have demonstrated that a CS over PMd can modulate contralateral M1 excitability, eliciting short-IHI at ISIs of 6 to 10 ms (Mochizuki et al. 2004; Koch et al. 2006; Ni et al. 2009) and long-IHI at ISIs of 40 to 60 ms (Ni et al. 2009). For PMv, a CS has been shown to elicit long-IHI and facilitation at other long-latency ISIs (Fiori et al. 2017), but short-IHI mechanisms from PMv remain unexplored. Previous investigations of PMd-to-M1 and PMv-to-M1 interactions have been conducted in separate studies and under different stimulation parameters (eg ISIs), precluding direct comparison between the two subregions and with M1-to-M1 IHI within a single study (Mochizuki et al. 2004; Ni et al. 2009; Fiori et al. 2017). Importantly, at ISIs as short as 4 to 5 ms, contralateral M1 facilitation (IHF) can occur, which is thought to represent the minimum transcallosal conduction time in humans (Hanajima et al. 2001; Ni et al. 2020). Below this minimum conduction latency, any modulation of contralateral M1 output likely reflects non-callosal contributions (ie subcortical or spinal pathways), a notion supported by non-human primate evidence demonstrating modulation of contralateral M1 during simultaneous stimulation (Quessy et al. 2016; Côté et al. 2017). Critically, non-callosal IHI, as probed by a 0 ms ISI (ie simultaneous CS and TS delivery), has not been examined from PMd or PMv in humans, despite evidence for modulation of contralateral M1 output in non-human primates (Quessy et al. 2016; Côté et al. 2017).

The present study was designed to systematically characterize inhibitory interactions between premotor cortex (PMd, PMv) and contralateral M1 using complementary TMS paradigms, integrating both single-pulse (iSP) and dual-site TMS (short-, long-, and non-callosal IHI) within the same study. By examining these interactions across multiple measures within the same participants, we sought to provide a more integrated account of premotor–motor connectivity than has been possible in prior work. Therefore, the objective of this study was to characterize the differential interhemispheric influence of PMd and PMv on contralateral M1 output using two complementary TMS approaches. Here, we assessed the iSP to index overall transcallosal inhibition from PMd, PMv and M1, and employed dual-site TMS to examine short- (10 ms ISI), long- (50 ms ISI), and non-callosal (0 ms ISI) IHI mechanisms. We hypothesized that an iSP would be elicited from PMd and PMv with unique silent period parameters compared to each other and to M1. We also hypothesized that PMd and PMv would display distinct short-, long- and non-callosal-IHI profiles compared to each other and to M1 itself. Finally, based on previous findings from M1 (Chen et al. 2003), we expected that iSP duration would be uniquely associated with long-IHI when elicited from PMd and PMv.

## Materials and methods

### Participants

Thirty right-handed adults (15 F, 18 to 39 years, mean age 26.4 [4.27]) took part in the study. Handedness was confirmed by using the Edinburgh Handedness Inventory (Oldfield 1971). A sensitivity analysis conducted using G^*^Power indicated that the current study had 90% power to detect an effect size f = 0.26 (equivalent to ~*η^2^_p_* = 0.06), which corresponds to a medium effect size at an alpha level of.05 (Lakens 2022). Informed consent was obtained before participation in any experimental protocol. Participants were screened for any potential contraindications to TMS and magnetic resonance imaging (MRI) using standard screening forms prior to participation. Participants reported no neurological conditions or disorders. The experimental procedures for this study were approved by the Ethics Committee of the Centre de recherche de l'Institute Universitaire de Gériatrie de Montréal (CRIUGM) under the approval number of “CER VN 21-22-36”.

### Experimental design

This study used a within-subject design that included one preliminary visit and two experimental sessions involving different TMS assessments. The preliminary visit included acquisition of a T1-weighted structural MRI scan used for TMS targeting and position monitoring. The first experimental session included the assessment of transcallosal inhibition, via measurement of the iSP, elicited from right hemisphere M1, PMd and PMv. See *Experimental Session 1: iSP evaluation* for further details. The second experimental session included the use of dual-site TMS to assess the neurophysiological mechanisms of short-, long- and non-callosal-IHI as elicited from right hemisphere M1, PMd and PMv to left M1 with three distinct ISIs (0, 10, and 50 ms; Fig. 1). See *Experimental Session 2: dual-site IHI evaluation* for further details. To accommodate diurnal fluctuations in M1 excitability (Merrell et al. 2024), the two experimental sessions were scheduled at the same time of day (±3 h) for each participant (Tamm et al. 2009).

**Figure 1:**
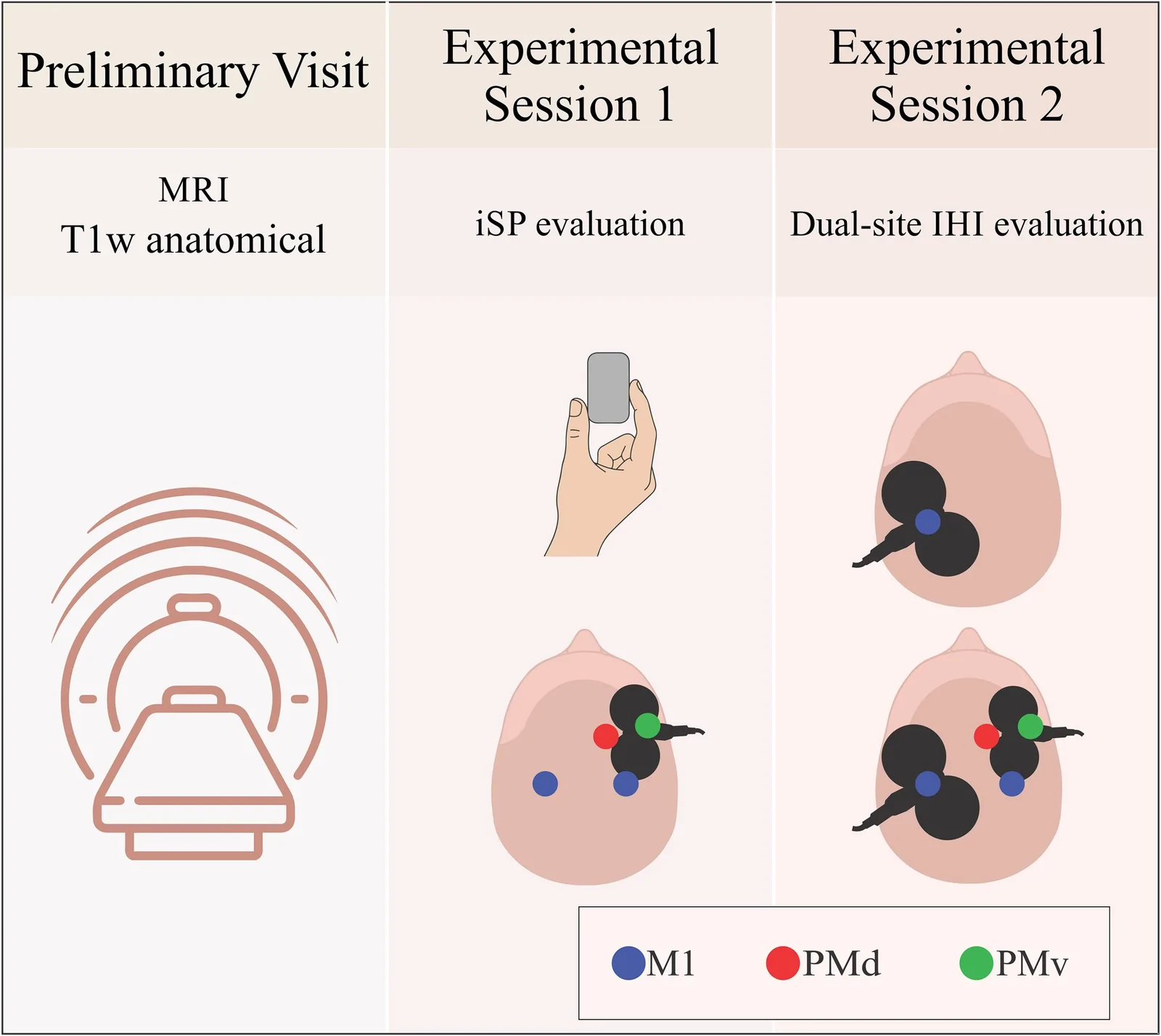
Experimental design. The study employed a within-subject design consisting of one preliminary visit and two experimental sessions. *Preliminary visit*: a T1w structural MRI scan was acquired for neuronavigation-guided transcranial magnetic stimulation (TMS) targeting and coil position monitoring. *Experimental session 1*: transcallosal inhibition was assessed via the ipsilateral silent period (iSP), elicited by suprathreshold single-pulse TMS over right hemisphere M1, PMd, and PMv while holding an isometric pinch grip contraction using the right hand (ipsilateral to the TMS pulse) at 50% of maximum voluntary contraction. *Experimental session 2*: dual-site TMS evaluated short-, long-, and non-callosal-interhemispheric inhibition (IHI), with conditioning stimuli (CS) over right hemisphere M1, PMd, and PMv and the test stimulus (TS) over left M1, at three ISIs (0, 10, and 50 ms). Colored circles indicate TMS target locations: M1 (blue), PMd (red), and PMv (green). Both experimental sessions were conducted at the same time of day (±3 h).

#### Electromyographic recording

Electromyographic (EMG) data were collected with 1-cm diameter surface electrodes from the first dorsal interosseous (FDI) muscle of both hands. Electrodes were placed over the FDI muscle in a belly-tendon configuration, with the ground electrode positioned over the ulnar styloid process. EMG signals were recorded using LabChart software (LabChart 8.0) with a PowerLab (PL3516 PowerLab, 16/35 16 Channel Recorder, AD Instruments, Colorado Springs, CO, United States) data acquisition system and amplified with a Dual Bio Amp (AD Instruments, Colorado Springs, CO, United States). Signals were sampled at 2 kHz and band-pass filtered (20 to 400 Hz) to minimize low-frequency movement artifacts and high-frequency noise, with an additional 50 Hz notch filter applied to reduce line noise. Data were captured in 500-ms sweeps (100 ms pre-stimulus to 400 ms post-stimulus). Trials were visually inspected offline, and any trials containing pre-stimulus EMG activity or artifacts were excluded from further analysis, ensuring that only trials with stable baseline EMG activity were retained.

#### Transcranial magnetic stimulation

During the TMS assessments, participants were comfortably seated on an adjustable chair and remained at rest, unless otherwise specified. TMS was delivered using two Magstim 200^2^ stimulators (Magstim, Whitland, United Kingdom) in a dual-coil configuration, with each stimulator connected to its own coil. This configuration was used for both single-pulse and dual-site TMS protocols. The BiStim^2^ module was not used to couple pulses; instead, it was retained in a decoupled (dual-coil) setup. The TS was delivered using a 7-cm outer-diameter figure-of-eight coil (Magstim 70 mm P/N 9790, Magstim Co., United Kingdom) over left M1. The CS was delivered over right hemisphere sites (PMd, PMv, or M1, depending on the condition) using a 5-cm outer-diameter branding-iron coil (Magstim Co., United Kingdom). For the iSP assessments, the TMS pulse was delivered using this same branding-iron coil connected to its respective Magstim 200^2^ stimulator. The TMS pulses generated were monophasic and with current flow in the posterior-to-anterior direction over left M1, and lateral-to-medial over right M1. The 7-cm outer diameter coil was oriented ~45° to the mid-sagittal line with the handle facing posteriorly over left M1. The 5-cm outer diameter branding iron coil was oriented perpendicular to the mid-sagittal over right M1. To ensure accurate and consistent coil placement, we used individual participant anatomical MRI (T1-weighted) for real-time position monitoring and cortical region targeting using the BrainSight neuronavigation system (Rogue Research Inc., Montreal, QC, Canada). Positioning the respective coils over left and right M1, the “hotspot” for the M1 FDI representation was identified, which was determined as the cortical location over M1 that elicited the largest and most consistent motor evoked potentials (MEPs) in contralateral FDI. For the right M1 hotspot, resting motor threshold (RMT) was determined as the lowest stimulator output to elicit peak-to-peak MEPs of at least 50 μV in 5 out of 10 consecutive trials (Rossini et al. 1994). During dual-site TMS, the stimulus intensity was adjusted to elicit consistent peak-to-peak MEP amplitudes of ~1 mV from left and right M1, which were used for our test (TS) and CS intensities, respectively (Daskalakis et al. 2002). We defined PMd as 2 cm anterior and 1 cm medial relative to the right M1 hotspot (O'Shea et al. 2007; Ni et al. 2009), and PMv as 3 cm anterior and 2.5 cm lateral relative to the right M1 hotspot (Bäumer et al. 2009). Additionally, using individual MRI neuronavigation, we ensured that the CS coil for PMd and PMv were over a gyrus rather than sulcus, slightly adjusting the CS coil placement accordingly (Ni et al. 2009). The Montreal Neurological Institute (MNI) coordinates for left M1, as well as right M1, PMd and PMv were recorded for each participant. These MNI coordinates were used for cortical target estimations, described further below.

### Experimental session 1: ipsilateral silent period evaluation

Experimental session 1 investigated iSP via the use of single-pulse TMS over right M1, PMd and PMv. The iSP was measured while participants held an isometric contraction of 50% maximum voluntary contraction of the right FDI with a pincer grip using a hand-held dynamometer, which was connected to a PowerLab data acquisition system that provided real-time visual feedback using a custom program with LabChart software (LabChart 8.0). 20 single TMS pulses were delivered at 150% RMT using the 5-cm outer diameter branding iron coil (Magstim Co., United Kingdom) over right M1, PMd and PMv individually (3 regions, 20 pulses/region = 60 pulses total) (Jung and Ziemann 2006; Neva et al. 2016). Participants were given rest periods of 30 to 60 s after collection of 10 trials with the purpose of avoiding fatigue.

### Experimental session 2: dual-site interhemispheric inhibition evaluation

In Experimental session 2, we further investigated the underlying mechanisms of IHI as assessed with dual-site TMS. The stimulation intensity required to elicit a ~1 mV MEP from the left M1 hotspot was used as the TS intensity and the intensity required to elicit ~1 mV MEP from the right M1 hotspot was applied to right PMd and PMv as the CS intensity. Dual-site IHI assessment consisted of delivering a CS over right PMd/PMv/M1 followed by a TS over left M1 at different ISIs (Ni et al. 2009; Neva et al. 2015; Fiori et al. 2017). Three distinct ISIs between CS and TS were evaluated: 0, 10, and 50 ms. These ISIs were chosen to investigate the influence of PMd, PMv and M1 on contralateral M1 excitable output via short (10 ms)-, long (50 ms)-, and non-callosal (0 ms)-IHI mechanisms (Ni et al. 2009; Côté et al. 2017). During IHI assessments elicited from PMd and PMv, we ensured that MEPs were not elicited from the CS in the contralateral FDI. To do this, when we observed MEPs from contralateral FDI, we systematically decreased the CS intensity in steps of 2% MSO until there were no observable MEPs. For PMd, one single participant displayed MEPs in contralateral FDI and the %MSO was decreased by a total of 6% MSO. No MEPs were observed in the contralateral FDI when applying the CS over PMv. Ten CS + TS trials and 10 TS alone trials were recorded for each cortical region (PMd, PMv and M1) and each ISI (0, 10 and 50 ms; 90 CS + TS pulses and 90 TS pulses = 180 total pulses).

### Data processing and statistical analysis

Repeated measures analysis of variance (RM-ANOVA) was used to analyze the data from Experimental session 1 (iSP data) and 2 (dual-site IHI data). The specific analyses will be described below in more detail. Residual statistics, skewness, and kurtosis values, along with plots, were generated to assess the normality and homoscedasticity of the data. Normality of data was further evaluated using the Shapiro–Wilk test with a significance level set at *P* < 0.001 (Gamst et al. 2008). To satisfy the normality assumption, the Greenhouse–Geisser correction was applied when sphericity was violated for RM-ANOVAs (variables: M1 0 ms and PMd 0 ms: W_30_ ≥ 0.683, ps ≥ 0.001) and data were square root-transformed for paired t-tests (variables: M1 at 0 and 10 ms, PMd at 0 ms, PMv at 0,10 and 50 ms; W_30_ ≥ 0.639, ps ≥ 0.001). Untransformed data are displayed in figures. Post hoc analyses were performed using Tukey’s HSD as appropriate. Effect sizes were calculated and reported as partial eta squared (*η^2^_p_*) following established guidelines for interpretation (Cohen 2013), where 0.01 indicates a small effect, 0.06 a moderate effect, and 0.14 a large effect. Statistical procedures were conducted by SPSS (SPSS 28.0) software. All data are reported as mean (SD) unless otherwise stated. Statistical significance was set at *P* < 0.05.

*EMG data processing for MEP amplitudes, iSP and IHI.* For iSP and IHI data, all trials were screened for unwanted voluntary muscle activity in the muscle(s) required to be at rest. Specifically, the pre-stimulus EMG (100 ms preceding TMS) of the resting muscle was inspected: the non-dominant (left) FDI during iSP assessment, and both FDIs during IHI assessment. Any trial showing visible voluntary activity (ie any clear departure from a stable quiescent baseline) in a muscle that should have been at rest was excluded from analysis (constituting 0.52% of trials). The actively contracting FDI during Experimental Session 1 was, by design, tonically active and was therefore not subject to this rest criterion. Specific data processing and analysis for Experimental sessions 1 and 2 are detailed further below.

*Baseline neurophysiological data: TS %MSO and TS MEP amplitudes*. Stable TS %MSO values during IHI assessment across cortical regions were ensured using a one-way RM-ANOVA, considering within-subject factor REGION (PMd, PMv, M1). Stable TS MEP amplitudes during IHI assessment across cortical regions were also ensured using one-way RM-ANOVA, considering within-subject factor REGION (M1, PMd, PMv).

*MNI Coordinate Estimation.* To estimate the anatomical location of premotor TMS targets, group-average MNI coordinates were analyzed using two complementary approaches. First, the SPM Anatomy Toolbox v3.0 (Eickhoff et al. 2005) was used to assess localization to cortical gyri and Brodmann areas based on probabilistic cytoarchitectonic maps. Second, Neurosynth (Yarkoni et al. 2011), a meta-analytic database of over 14,000 functional magnetic resonance imaging studies, was used to evaluate functional associations with premotor terms. Additionally, anatomical separation between targets was quantified by calculating Euclidean distances in MNI space. Specifically, PMd-to-M1 distance was calculated to assess spatial distinctness from M1 representation, and vertical (superior–inferior) separation between PMd and PMv was calculated to assess distinction between dorsal and ventral premotor areas. Recorded z-coordinates were reduced by 20 mm to account for scalp-to-cortex distance prior to anatomical analysis, consistent with previous work (Opitz et al. 2011).

*Experimental Session 1*. Transcallosal inhibition elicited from the premotor cortices (PMd and PMv) and M1 was quantified by the iSP. The iSP was defined as the transient reduction in volitional EMG activity elicited by TMS applied over the cortex ipsilateral to the active muscle (Fling and Seidler 2012; Borich et al. 2015). To calculate the iSP as elicited from each region (PMd, PMv and M1), EMG data were full-wave rectified and averaged for each participant. Pre-stimulus mean EMG (pre-stimulus_mean_) was defined as the voluntary EMG signal recorded 100 ms prior to TMS delivery. The iSP onset (iSP_onset_) was defined as the post-TMS time point (in ms) where the rectified EMG fell below the pre-stimulus_mean_ EMG signal and continued to decrease to less than 2 standard deviations below this level. The iSP offset (iSP_offset_) was defined as the point at which the EMG signal resumed the level of the pre-stimulus_mean_ activity consistently for a minimum of 2 ms (Jung and Ziemann 2006; Fling and Seidler 2012). The iSP duration comprised all data points between iSP_onset_ and iSP_offset_ (calculated in ms). The average rectified EMG level during the iSP was defined as the iSP mean (iSP_mean_) (Jung and Ziemann 2006). The iSP_mean_ was then expressed as a ratio of the mean pre-stimulus EMG (iSP_mean_/pre-stimulus_mean_), where a lower value indicates more inhibition. Custom MATLAB scripts (Mathworks, Natick, MA) were used to identify parameters of the iSP for each participant (Mang et al. 2015; Neva et al. 2016). While collecting the iSP elicited from PMd, we observed the appearance of contralateral MEPs. We thus quantified the mean peak-to-peak MEP amplitudes from PMd and compared them to those elicited from M1. No MEPs were elicited during iSP elicited from PMv.

Our first analysis quantified whether a significant iSP was elicited following stimulation over PMd, PMv and M1. To do this, we compared the iSP_mean_ to the pre-stimulus_mean_ EMG using a two-way RM-ANOVA with within-subjects factors EMG-SIGNAL (iSP_mean_, pre-stimulus_mean_) and REGION (PMd, PMv, M1). Our second analysis further quantified iSP parameters (iSP_mean_/pre-stimulus_mean_, iSP_onset_ and iSP duration), performing one-way RM-ANOVAs using the within-subjects factor REGION (PMd, PMv, M1). Finally, paired t-tests were performed comparing the mean peak-to-peak MEP amplitudes elicited from PMd and M1 during iSP collection.

*Experimental Session 2*. Dual-site paired-pulse TMS was used to assess short-, long- and potential non-callosal mechanisms of IHI from the right premotor cortices and M1 to left M1. Custom MATLAB scripts were used to assess peak-to-peak MEP amplitudes (measured in millivolts, mV) as elicited by TS alone and CS + TS for each cortical region. IHI magnitude was quantified as a ratio of CS + TS MEP amplitude over TS MEP amplitude (Kujirai et al. 1993): IHI ratio = (CS + TS)/TS, where smaller values indicate greater inhibition, while larger values indicate less inhibition (disinhibition or facilitation). This was performed for each cortical region (PMd, PMv, M1) and ISI (0, 10, and 50 ms). The ISIs were specifically chosen to investigate short (10 ms)- and long (50 ms)-, and potential non-callosal (0 ms)-IHI mechanisms impacting contralateral M1 output (Mochizuki et al. 2004; Ni et al. 2009; Côté et al. 2017; Fiori et al. 2017).

Our first analysis assessed inhibition of left M1 output elicited by a CS over right hemisphere PMd, PMv or M1. To do this, we compared peak-to-peak MEP amplitudes elicited with TS alone over left M1 to those elicited from CS + TS. Paired t-tests compared MEP amplitude elicited with a TS alone to CS + TS within each cortical region across ISIs (10, 50, and 0 ms; Bonferroni correction applied as appropriate; (Ni et al. 2009). Our second analysis assessed IHI magnitude between cortical regions and ISIs using a two-way RM-ANOVA involving within-subjects factors REGION (PMd, PMv, M1) and ISI (0, 10, and 50 ms), using IHI ratio as the dependent measure.

*Correlational analyses of Experimental Session 1 (iSP) and 2 (IHI) data*. Bivariate correlational analyses (Pearson’s r_p_ for normally distributed data or Spearman’s r_s_ for not normally distributed data) were conducted to characterize the strength in relationships between iSP duration and long-IHI (ie 50 ms ISI). As previous research has shown that M1 iSP duration was associated with M1 IHI at longer ISIs (eg 40 ms) but not shorter ISIs (Chen et al. 2003), we examined whether PMd/PMv demonstrated similar associations.

## Results

### Baseline neurophysiological data

For TS %MSO values during IHI assessments, there was no significant main effect of REGION (F_2, 58_ = 0.222, *P* = 0.802, *η^2^_p_* = 0.008), confirming stable TS intensity during assessment of IHI across the different brain regions examined. For TS MEP amplitudes during IHI assessments, there was no significant main effect of REGION (F_2, 58_ = 0.125, *P* = 0.882, *η^2^_p_* = 0.004), confirming consistent corticospinal output excitability throughout the assessment of IHI across the different brain regions (see Supplementary Table 1).

### Montreal Neurological Institute (MNI) coordinate estimation

Group-average MNI coordinates (x, y, z) for the TMS targets were: right M1 (47.5, −10.2, 59.5), right PMd (40.4, 11.2, 57.7), and right PMv (61.8, 12.4, 28.5). SPM Anatomy Toolbox analysis localized the PMd coordinates to the middle frontal gyrus (macroanatomy: 66%), positioned immediately anterior to the cytoarchitectonic border of Area 6. The PMv coordinates were localized to the ventral precentral gyrus/Area 44 transition zone (cytoarchitecture: Area 44, 15.2%; macroanatomy: precentral gyrus, 31%). Neurosynth functional decoding revealed strong associations with premotor function for both targets (PMd: posterior probability = 0.60 for “dorsal premotor cortex”; PMv: posterior probability = 0.56 for “ventral premotor”). Euclidean distance between the PMd target and the M1 hotspot was 22.6 mm, with the primary separation along the anterior–posterior axis (Δy = 21.4 mm). The vertical separation between PMd and PMv targets was 32.7 mm (Δz), suggesting distinct dorsal and ventral premotor localizations.

### Experimental session 1: Ipsilateral silent period elicited from dorsal and ventral premotor cortices and primary motor cortex 

Figure 2 displays representative data of individual EMG traces, mean and individual data demonstrating iSPs elicited from M1, PMd and PMv. Two-way RM-ANOVA revealed a significant interaction between EMG-SIGNAL and REGION (F_2, 58_ = 9.264), *P* < 0.001, *η^2^_p_* = 0.242; Fig. 2A and B). Post hoc analyses demonstrated a significant decrease in rectified EMG during the iSP_mean_ compared to the pre-stimulus_mean_ for PMd (*P* < 0.001), PMv (*P* < 0.001) and M1 (*P* < 0.001; Fig. 2). Main effects of EMG-SIGNAL (F_1, 29_ = 110.560, *P* < 0.001, η*^2^_p_* = 0.792) and REGION (F_2, 58_ = 6.043, *P* = 0.004, *η^2^_p_* = 0.172; Fig. 2A and B) were also found.

**Figure 2:**
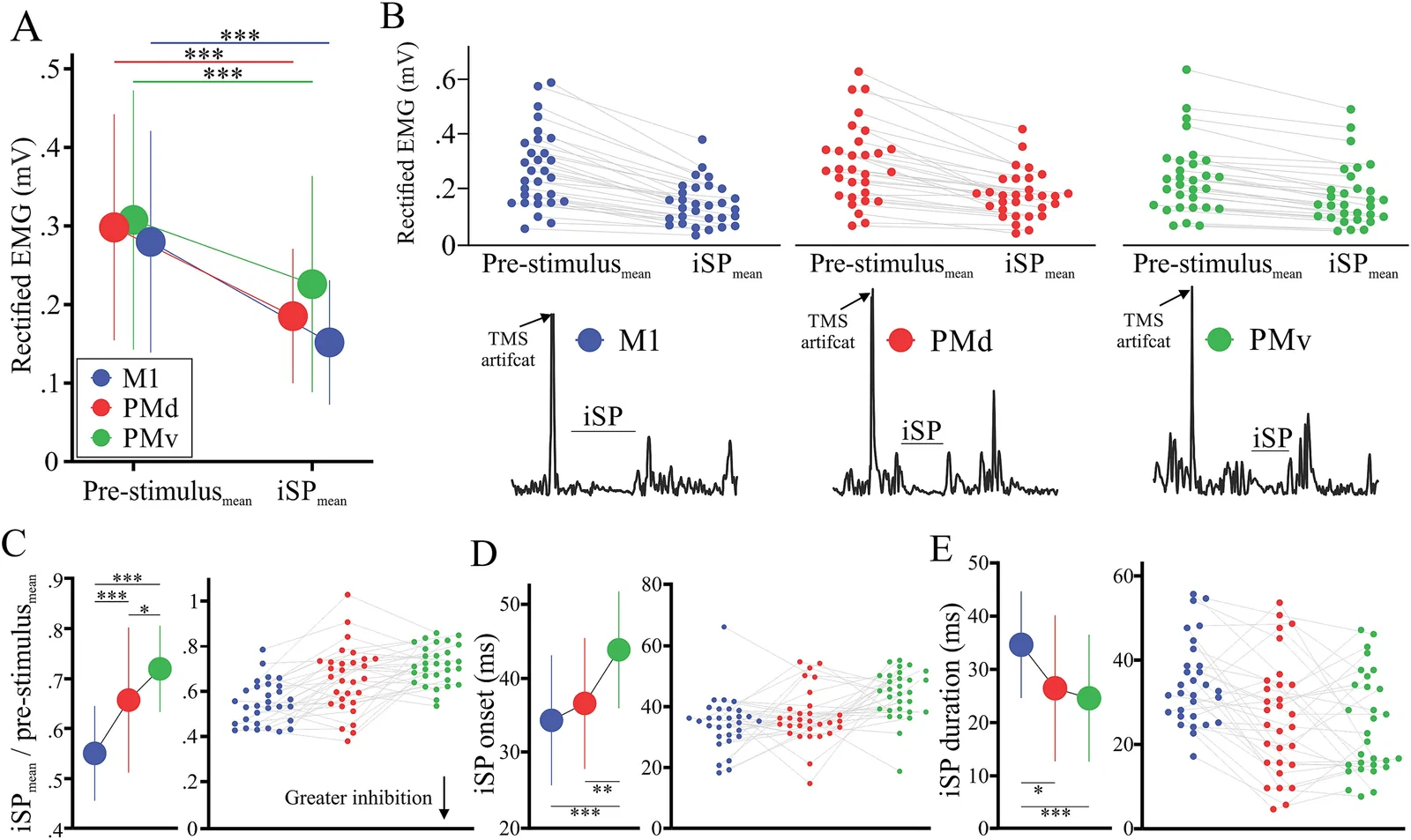
Experimental session 1: iSP results. (A) Group mean (± SD) rectified EMG amplitude (mV) for the pre-stimulus period and iSP window across stimulation regions (M1, blue; PMd, red; PMv, green). (B) *Top*: Individual participant data showing rectified EMG amplitude during the pre-stimulus period and iSP window for M1, PMd, and PMv, with gray lines connecting paired observations. *Bottom*: Representative single-participant EMG traces illustrating the iSP following suprathreshold TMS over M1, PMd, and PMv, with the TMS artifact and iSP window indicated. (C) iSP magnitude, expressed as the ratio of mean EMG during the iSP to pre-stimulus mean EMG (iSP_mean_/pre-stimulus_mean_), shown as group means ± SD (left panel) and individual participant data with connected observations (right panel). Lower values indicate greater inhibition. (D) iSP onset latency (ms) shown as group means ± SD (left panel) and individual participant data (right panel). (E) iSP duration (ms) shown as group means ± SD (left panel) and individual participant data (right panel). In all panels, asterisks denote significant post hoc differences (^*^  *P* < 0.05, ^**^  *P* < 0.01, ^***^  *P* < 0.001).

For iSP_mean_/pre-stimulus_mean_ one-way RM-ANOVA revealed a significant main effect of REGION (F_2, 58_ = 29.127, *P* < 0.001, *η^2^_p_* = 0.501; Fig. 2C), with post hoc analyses showing that M1 (*P* < 0.001) displayed the greatest magnitude of inhibition, followed by PMd (*P* < 0.001), then PMv (*P* = 0.01). For iSP_onset_, one-way RM-ANOVA revealed a significant main effect of REGION (F_2, 58_ = 10.229, *P* < 0.001, *η^2^_p_* = 0.261; Fig. 2D), with post hoc analyses showing both M1 (*P* < 0.001) and PMd (*P* = 0.004) displayed shorter iSP onsets compared to PMv, with no difference between M1 and PMd (*P* = 0.550). Finally, for iSP duration, one-way RM-ANOVA revealed a significant main effect of REGION (F_2, 58_ = 5.976, *P* = 0.004, *η^2^_p_* = 0.121; Fig. 2E), with M1 displaying significantly longer duration than PMd (*P* = 0.026) and PMv (*P* = 0.005), while PMd and PMv demonstrated similar durations (*P* = 0.819).

*Assessment of MEPs elicited in contralateral (left) FDI during iSP*. A paired *t-*test demonstrated that the peak-to-peak MEP amplitude elicited when assessing iSPs from PMd and M1 was significantly different (*P* < 0.001). Specifically, peak-to-peak MEP amplitudes elicited from M1 (5.1 ± 2.3 mV) were significantly greater than those elicited from PMd (2.9 ± 2.1 mV). No MEPs were observed when assessing iSP elicited from PMv.

### Experimental session 2: dual-site interhemispheric inhibition assessment from dorsal and ventral premotor cortices and primary motor cortex 

Figure 3 displays representative data of individual EMG traces, mean and individual data of MEPs elicited from TS alone and CS + TS as well as IHI ratio data from M1, PMd and PMv for each ISI. For the 10 ms ISI, paired t-tests showed significant decrease in MEP amplitude as elicited from the CS + TS compared to TS alone for PMd (*P* < 0.001) and M1 (*P* = 0.001), but not PMv (*P* = 0.886; Fig. 3B). For the 50 ms ISI, paired t-tests showed MEP amplitude decrease as elicited from CS + TS compared to TS alone for PMd (*P* < 0.001), M1 (*P* = 0.011) and PMv (*P* = 0.007; Fig. 3C). Finally, for the 0 ms ISI, paired t-tests showed significant reductions in MEP amplitude as elicited from CS + TS compared to TS alone for PMd, PMv and M1 (ps < 0.001; Fig. 3A).

**Figure 3:**
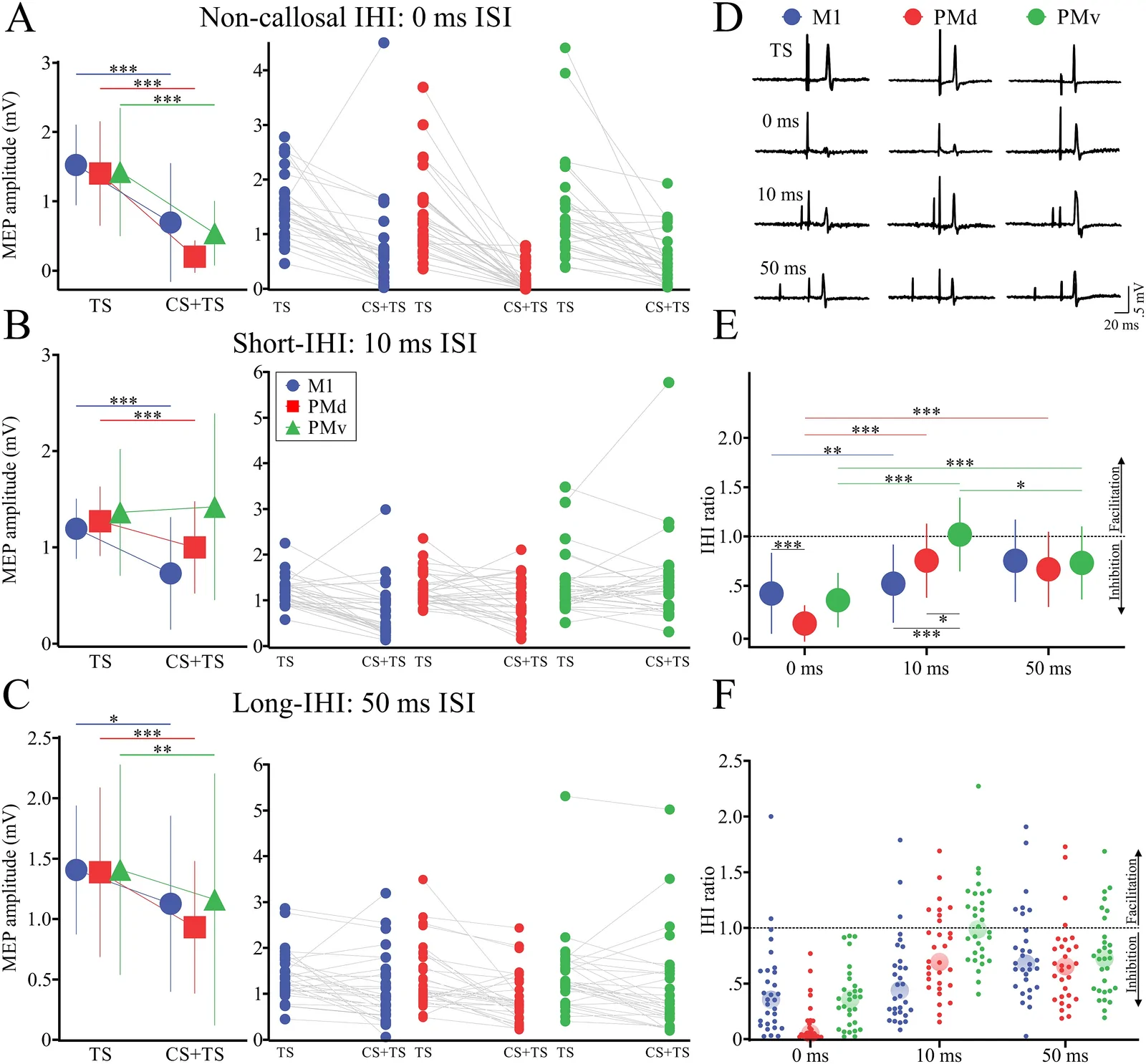
Experimental session 2: IHI results. (A–C) *Left panels*: Group mean (± SD) MEP amplitude (mV) for the test stimulus alone (TS) and the conditioned stimulus paired with the test stimulus (CS + TS) for M1 (blue circles), PMd (red squares), and PMv (green triangles), at ISIs of 0 ms (A: Non-callosal IHI), 10 ms (B: Short-IHI), and 50 ms (C: Long-IHI). *Right panels*: Individual participant MEP amplitudes for TS and CS + TS across all three stimulation sites at each ISI, with gray lines connecting paired observations. (D) Representative single-participant EMG traces for TS alone and CS + TS conditions at each ISI (0, 10, and 50 ms), shown separately for each conditioning site (M1, PMd, PMv). (E) Group mean (± SD) and (F) individual participant IHI ratios (CS + TS MEP/TS MEP) across ISIs for M1, PMd, and PMv. The dashed horizontal line at 1.0 demarcates inhibition (below) from facilitation (above). In all panels, asterisks denote significant post hoc differences (^*^  *P* < 0.05, ^**^  *P* < 0.01, ^***^  *P* < 0.001).

Figure 3E and F displays mean and individual IHI ratio data. Two-way RM-ANOVA revealed a significant interaction between REGION and ISI (F_3.34, 96.74_ = 8.895, *P* < 0.001, *η^2^_p_* = 0.235). For the 10 ms ISI, post hoc analyses demonstrated that M1 (*P* < 0.001) and PMd (*P* = 0.048) displayed greater inhibition than PMv, with no difference between M1 and PMd (*P* = 0.119). For the 50 ms ISI, there were no differences between cortical regions (ps > 0.980). For the 0 ms ISI, post hoc analyses demonstrated that PMd displayed greater inhibition compared to M1 (*P* = 0.001), with no other differences (ps > 0.955). Within PMd, post hoc analyses demonstrated greater inhibition with a 0 ms compared to both 10 (*P* < 0.001) and 50 ms (*P* < 0.001) ISIs, with no difference between 10 and 50 ms (*P* = 0.977) ISIs. Within PMv, post hoc analyses demonstrated greater inhibition with a 0 ms ISI compared to both 10 and 50 ms ISIs (ps < 0.001), along with greater inhibition elicited with a 50 ms compared to 10 ms ISI (*P* = 0.021). Within M1, post hoc analyses demonstrated greater inhibition elicited with a 0 ms compared to a 50 ms (*P* = 0.003) ISI, with no other differences (ps > 0.125). We also observed main effects of ISI (F_1.99, 57.57_ = 48.134, *P* < 0.001, *η^2^_p_* = 0.624) and REGION (F_1.79, 51.88_ = 6.812, *P* = 0.002, *η^2^_p_* = 0.190).

### Correlational analyses between ipsilateral silent period and interhemispheric inhibition ratio data

Figure 4 displays correlational analysis between iSP duration and long-IHI tested with a 50 ms ISI for each region. We found a significant negative correlation for PMd (r_s_ = −0.404, R^2^ = 0.163, *P* = 0.027) and PMv (r_s_ = −0.365, R^2^ = 0.133, *P* = 0.047), while we did not for M1 (r_s_ = −0.021, R^2^ = 0.0004, *P* = 0.912).

**Figure 4:**
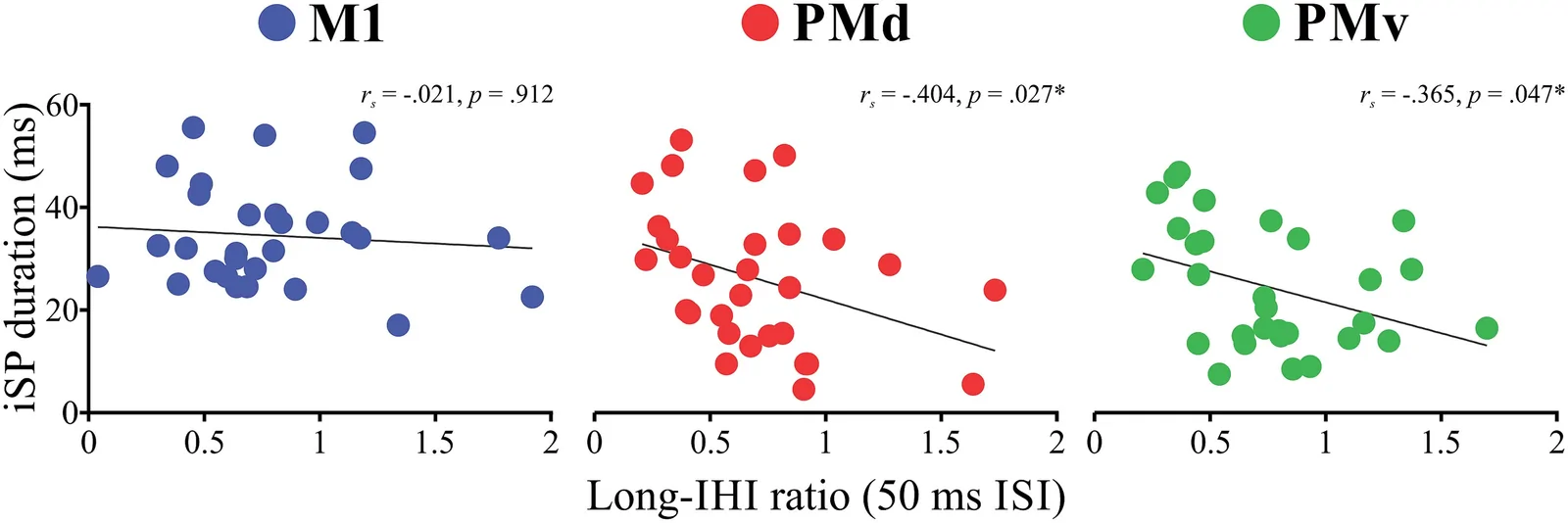
Correlations between iSP duration and long-IHI ratio. Scatterplots illustrating the relationship between iSP duration (ms; Experimental session 1) and the long-IHI ratio (50 ms ISI; Experimental session 2) for M1 (blue, *left*), PMd (red, *middle*), and PMv (green, *right*). Each data point represents an individual participant. Spearman’s rank correlation coefficient (${r}_s$) and corresponding *p* value are displayed in each panel. Asterisks (^*^) denote statistically significant correlations (*P* < 0.05).

## Discussion

The present findings provide a systematic characterization of interhemispheric interactions between premotor cortices and contralateral M1 across complementary TMS paradigms. Three primary novel findings emerged from our approach: (i) we demonstrate that unique iSP properties can be elicited from both PMd and PMv, extending this measure beyond its more established application in M1; (ii) we observe non-callosal IHI at a 0 ms interval across regions, suggesting contributions from mechanisms that extend beyond classical transcallosal pathways; (iii) we also provide a first characterization of IHI from PMv at a 10 ms ISI. In addition, PMd and PMv displayed greater diversity in their inhibitory profiles across short-, long- and non-callosal-IHI in comparison to M1, suggesting that these PMC subregions exhibit distinct modulation patterns both relative to each other and to M1. Finally, iSP duration correlated with long-IHI magnitude for PMd and PMv, linking these two measures and suggesting they reflect partly overlapping neural mechanisms. Collectively, these findings suggest that premotor cortices can be dynamic contributors to interhemispheric interactions with M1, complementing our understanding of M1-mediated pathways. These findings may have implications for motor recovery following stroke, where premotor cortices can be recruited into reorganized motor networks, and altered PMC-M1 interactions may contribute to the state of motor deficits. The distinct inhibitory profiles observed across premotor regions suggest that these areas may represent differentiated targets for neuromodulation approaches targeting interhemispheric interactions within motor-related networks. Here, we will discuss the potential explanations for, and mechanisms supporting, our findings.

### PMd and PMv elicit distinct ipsilateral silent periods

To our knowledge, the iSP has only been elicited from M1 in healthy and clinical populations. Here, we demonstrated that PMd and PMv also elicit iSPs, with largely unique silent period properties distinct from M1 and from each other (see *Results* for details). Importantly, these findings indicate that transcallosal inhibition is not a unitary process defined by M1, but varies depending on the cortical region stimulated, as reflected by distinct iSP properties across premotor and motor areas. Our demonstration of an iSP elicited from PMd and PMv extends prior dual-site TMS studies showing IHI from these premotor regions to contralateral M1 (Mochizuki et al. 2004; Ni et al. 2009; Fiori et al. 2017), suggesting both techniques may engage shared transcallosal inhibitory pathways, albeit across different temporal windows. Our findings extend earlier dual-site TMS work which showed that PMd and PMv exhibit distinct inhibitory profiles compared to M1 (Ni et al. 2009; Fiori et al. 2017). Notably, Ni et al. (2009) found that PMd-elicited IHI emerged at fewer interstimulus intervals compared to M1-elicited IHI, with a slight shift toward longer intervals in long-latency IHI (Ni et al. 2009). Our iSP results parallel these observations: PMd showed reduced iSP magnitude compared to M1, consistent with comparatively less engagement of transcallosal inhibitory pathways. PMd onset latencies, however, were comparable to M1, suggesting that while inhibitory strength differs, transcallosal conduction timing between the two regions is similar. These parallels are meaningful given that iSP likely reflects long-IHI mechanisms (Chen et al. 2003), and Ni et al. demonstrated that PMd-elicited IHI was prominent at longer-latency ISIs (≥40 ms) (Ni et al. 2009). The longer iSP onset latency for PMv compared to M1 and PMd likely reflects its distinct terminal organization of corticospinal projections. PMv compared to PMd has fewer direct projections to the lower cervical enlargement, potentially requiring more complex and indirect pathways (Borra et al. 2010). In contrast, PMd has more robust (~5 times) corticospinal terminal projection to cervical enlargement (Morecraft et al. 2019). These differences between PMd and PMv may help explain shorter iSP onset latency from PMd. Together, these findings suggest that PMd may differentially engage transcallosal pathways with distinct temporal dynamics compared to M1, which can be assessed via the iSP using single-pulse TMS.

A critical methodological consideration in this study is whether the iSP elicited from premotor regions truly reflects PMC-to-M1 inhibition or from the current spread/indirect activation of ipsilateral M1. Notably, contralateral MEPs were observed during PMd stimulation, indicating that the TMS pulse may have activated ipsilateral M1, either via current spread or secondary activation. This raises the possibility that the resulting iSP reflects, at least in part, transcallosal inhibition via M1. However, these MEPs were markedly smaller than those evoked by direct M1 stimulation, suggesting that premotor regions contributed to the observed inhibition. In contrast, PMv stimulation did not elicit contralateral MEPs. Consistent with this interpretation, Ni et al. (2009) varied the CS position at a midpoint between M1 and PMd as a control location and reported evidence for differential activation of PMd and M1 even at suprathreshold intensities, indicating that premotor conditioning effects are not solely attributable to M1 co-activation (Ni et al. 2009). Several lines of evidence from our data support the notion that the iSPs elicited from PMd, PMv, and M1 each arise from anatomically and functionally distinct cortical sources. First, although significant reductions in ongoing EMG activity were observed from all three cortical stimulation sites, the iSP magnitude via normalized EMG followed a systematic gradient (M1 > PMd > PMv). Importantly, this gradient may also reflect differences in effective stimulation strength due to distance from M1, rather than solely site-specific physiological differences. If the PMd or PMv results were primarily due to current spread to or indirect activation of ipsilateral M1, we would typically expect the resulting iSP profiles to closely resemble those elicited from M1. Instead, PMd and PMv displayed unique iSP durations and the onset latency elicited from PMv was distinct from M1; while PMd onset latency was not statistically different, there was a slight delay compared to M1. Second, our correlational analyses revealed region-specific associations. We found that the iSP duration elicited from PMd was significantly correlated with the magnitude of long-latency IHI from PMd. The same correlational pattern was found for PMv. Crucially, these relationships were specific to PMd and PMv, with M1 not displaying this correlational pattern with long-IHI, in contrast to previous findings (Chen et al. 2003). Nevertheless, our findings suggest that the inhibitory circuits activated by single-pulse TMS over PMd and PMv may be distinct, at least in part, from those activated over M1. Third, MNI coordinate analysis demonstrated anatomical distinctness between stimulation sites. The PMd target was located 22.6 mm from the M1 hotspot, placing it in the middle frontal gyrus at the anterior border of Area 6 (Picard and Strick 2001; Chouinard and Paus 2006). This separation exceeds the estimated spread of TMS electric fields previously reported (~10 to 15 mm; (Thielscher and Kammer 2004)), providing some evidence of anatomical separation but does not exclude direct or indirect M1 activation. Furthermore, PMd and PMv were separated by 32.7 mm vertically (superior–inferior axis), with PMv localized to the ventral precentral gyrus/Area 44 transition zone, supporting anatomical separation between premotor regions (Picard and Strick 2001; Chouinard and Paus 2006). Collectively, while a current-spread contribution to the PMd iSP cannot be excluded, this converging evidence supports the use of the iSP as an index of PMC-to-M1 transcallosal inhibition, allowing the further distinction between interhemispheric connectivity from PMd and PMv to contralateral M1.

### PMd and PMv exhibit diverse interhemispheric inhibitory profiles

Our dual-site TMS results assessed the inhibitory profiles of PMd and PMv across different temporal windows, revealing that these premotor regions exerted differential influences on contralateral M1, while also having distinct patterns to M1-to-M1 IHI.

IHI elicited from PMd at short (10 ms) and long (50 ms) ISIs likely reflects distinct transcallosal cortico-cortical pathways (Ferbert et al. 1992; Irlbacher et al. 2007; Ni et al. 2009). The timing of short-IHI results is consistent with relatively fast transcallosal transmission, suggesting PMd projects to contralateral M1 either directly or via contralateral PMd. Previous studies demonstrated PMd-elicited short-IHI at 6, 8, and 10 ms ISIs (Mochizuki et al. 2004; Koch et al. 2006; Ni et al. 2009). While the specific pathway mediating short-IHI remains uncertain, M1-to-M1 short-IHI (6 to 12 ms ISIs) is thought to involve transcallosal signals interacting with intracortical circuitry in contralateral M1, potentially through mechanisms of short-interval intracortical inhibition (Daskalakis et al. 2002). Given that PMd and M1 produced similar degrees of short-IHI in our study, PMd-elicited inhibition may engage similar intracortical mechanisms in contralateral M1, though this needs to be tested in future work. In this context, shifts in interhemispheric PMC-M1 interactions may be particularly relevant following stroke, where imbalances within the motor-related networks are thought to influence recovery trajectories (Grefkes et al. 2008; Bestmann et al. 2010). Long-IHI (50 ms) from PMd aligns with previous findings showing that PMd modulates contralateral M1 output through slower, potentially GABA_B_-mediated intracortical circuits at ISIs of 40 to 60 ms (Ni et al. 2009). M1-elicited long-IHI (30 to 50 ms ISIs) is believed to involve polysynaptic circuits or additional intracortical loops beyond that of short-IHI (Udupa et al. 2010; Ghosh et al. 2013). It is possible that PMd-to-M1 and M1-to-M1 long-IHI share similar mechanisms due to the similar magnitude and relative timing of inhibition as shown with our dual-site TMS results. However, the slight shift toward longer intervals in long-latency IHI elicited from PMd compared to M1 found in previous work (Ni et al. 2009), along with differences in our iSP parameters elicited from PMd compared to M1, suggests potentially distinct pathways of connectivity between PMd and contralateral M1 compared to M1-to-M1 interactions; yet this warrants future investigation.

A notable finding was the robust inhibition elicited from PMd at the 0 ms ISI. Given the novelty of this finding, any mechanistic interpretation should be considered preliminary. Since 0 ms falls below the minimum conduction time for transcallosal communication in humans (~4 to 5 ms; (Hanajima et al. 2001; Ni et al. 2020)), this effect is unlikely to be mediated by cortico-cortical pathways. Instead, this non-callosal inhibition may reflect a convergence of descending volleys from PMd and contralateral M1 at subcortical and/or spinal levels. However, the precise mechanisms underlying this effect remain unclear. While differences in stimulation parameters and methodological approaches may contribute to discrepancies with prior animal work (Quessy et al. 2016; Côté et al. 2017), our data do not allow us to determine whether inhibitory or facilitatory pathways are preferentially engaged at these sites of convergence. One possible interpretation is that descending signals interact at subcortical or spinal loci, potentially including brainstem structures such as the reticular formation. Consistent with this possibility, animal work has shown that PMd sends dense corticoreticular projections to the pontomedullary reticular formation (Keizer and Kuypers 1989; Kably and Drew 1998; Fregosi et al. 2017). However, these interpretations remain necessarily speculative, and future studies are needed to directly test the underlying pathways and mechanisms of this non-callosal IHI. It is important to note that because significant inhibition was observed across all stimulation sites (ie PMd, PMv, and M1), a contribution from nonspecific or stimulation-related artifact cannot be excluded. While the greater inhibition observed from PMd relative to M1 is evidence against a uniform artifact, the absence of consistent differences across all regions warrants cautious interpretation.

In contrast to PMd, PMv exhibited a distinct inhibitory profile characterized by absent short-IHI and preserved long-IHI. PMv did not elicit significant inhibition at the 10 ms ISI, in contrast to PMd and M1. However, PMv produced long-IHI at 50 ms ISI, comparable in magnitude to PMd and M1. These findings align with Fiori et al. (2017), who demonstrated PMv-elicited long-latency IHI at 40 ms and 150 ms ISIs (Fiori et al. 2017). The absence of short-IHI combined with preserved long-IHI suggests that PMv influences contralateral M1 through slower, potentially polysynaptic pathways involving higher-level cortical (eg prefrontal and parietal cortices) or subcortical relays (eg thalamus, brainstem structures) rather than direct transcallosal projections (Fiori et al. 2017; Ruddy et al. 2017). Our iSP results provide support for this unique temporal signaling, where PMv showed significantly longer onset latency and reduced magnitude compared to PMd and M1. Our distinct iSP findings between premotor regions suggest that while PMv engages transcallosal pathways, these connections may be functionally distinct or less direct than those from PMd (Fiori et al. 2017; Ruddy et al. 2017). Importantly, the absence of short-IHI in our study does not rule out direct transcallosal PMv-to-M1 connectivity. Anatomical evidence confirms direct PMv projections to contralateral M1, particularly in rostral M1 where there is less digit representation (Dancause et al. 2007). Further, the difference in callosal fiber density from PMv and PMd to contralateral M1 is relatively small (Boussaoud et al. 2005), suggesting the functional distinctions observed here may reflect differences in pathway efficacy rather than anatomy alone. Collectively, these findings suggest that PMv-to-M1 interhemispheric communication operates through physiologically distinct mechanisms compared to PMd and M1 (Ruddy et al. 2017). Consistent with this, the absence of contralateral MEPs during PMv stimulation suggests that indirect M1 activation is less likely, although it cannot be entirely excluded. Future research combining neurophysiological and structural connectivity approaches would be beneficial to clarify these potential pathway differences.

PMv produced robust non-callosal IHI at 0 ms ISI, comparable in magnitude to PMd and M1. Our findings align with previous non-human primate work showing that simultaneous microstimulation of PMv and contralateral M1 primarily elicited inhibition of M1 output (Quessy et al. 2016; Côté et al. 2017). As with PMd, this effect may reflect convergence of descending volleys at subcortical locations, such as the reticular formation and medulla, as animal models demonstrate these anatomical connections (Kably and Drew 1998; Wise 2006), without discounting convergence at spinal loci. Although our results suggest that this inhibition may not be via transcallosal pathways, the specific loci and mechanisms underlying this interaction remain uncertain and cannot be determined from the present data.

### M1-to-M1 non-callosal interhemispheric inhibition

Our finding of robust inhibition with simultaneous bilateral M1 stimulation (0 ms ISI) is, to our knowledge, novel. Previous work has characterized distinct temporal phases of M1-to-M1 modulation: long-IHI at ~40 to 60 ms ISIs (Ni et al. 2009), short-IHI at ~10 ms ISIs (Hanajima et al. 2001; Daskalakis et al. 2002; Ni et al. 2009; Ni et al. 2020), facilitation at 4 to 5 ms ISIs likely reflecting initial excitatory transcallosal transmission (Hanajima et al. 2001; Ni et al. 2020), and no modulation at 1 to 3 ms ISIs likely representing intervals too brief for transcallosal propagation (Ni et al. 2020). Short- and long-IHI likely arise from interaction with GABAergic inhibitory interneurons that suppress pyramidal neuron output (Daskalakis et al. 2002; Hui et al. 2020; Ni et al. 2020). At 0 ms ISI, the observed inhibition exceeded M1-elicited long-IHI (50 ms) and was comparable to short-IHI (10 ms), though smaller in magnitude than PMd-elicited inhibition. Since simultaneous stimulation precludes a transcallosal route (Hanajima et al. 2001; Ni et al. 2020), this observation may reflect interactions between descending volleys at subcortical and/or spinal levels (Fisher et al. 2012; Fregosi et al. 2017). However, alternative explanations, including non-specific effects of simultaneous stimulation, cannot be excluded.

### Limitations

Certain limitations should be acknowledged when interpreting the findings of this study. First, iSP measurements were performed at a single TMS intensity (150% RMT) and contraction level (50% of maximum voluntary contraction), based on evidence that sufficiently high TMS intensities over M1 and contraction levels reliably elicit iSPs when only the ipsilateral muscle is activated (Chen et al. 2003; Jung and Ziemann 2006; Davidson and Tremblay 2013). However, there is evidence that lower TMS intensities can also elicit comparable iSP magnitudes from M1 (Chen et al. 2003). Future work should investigate whether PMd- and PMv-elicited iSPs demonstrate similar intensity- and contraction-dependent modulation as M1, including multiple contraction levels to assess potential ceiling effects. Second, the iSP assessment from PMd elicited contralateral MEPs, raising the possibility that current spread to, or indirect activation of, ipsilateral M1 partially contributed to the observed inhibition. However, these MEPs were significantly smaller than those elicited by direct M1 stimulation. Additional observations, including distinct PMd and M1 iSP parameters, significant PMv iSPs without contralateral MEPs, dual-site IHI intensities adjusted to avoid contralateral MEPs, and separation among the MNI coordinates of PMd, PMv, and M1, provide some support for premotor contributions (Opitz et al. 2011). Nevertheless, these observations cannot establish the spatial extent or regional specificity of stimulation. The present study would have been strengthened by individualized electric field modeling (eg SimNIBS). Future studies should incorporate individualized head models and examine stimulation intensity, target location, and coil current orientation to more directly assess current spread and its contribution to premotor stimulation effects. Third, our study would have benefited from examining additional ISIs and conditioning stimulus current directions. Shorter ISIs (4 to 5 ms) would clarify whether PMd or PMv elicit facilitation similar to M1 at these latencies (Hanajima et al. 2001; Ni et al. 2020). Intermediate ISIs for PMd (between 10 and 50 ms) and longer ISIs for PMv (80 and 150 ms) would provide more complete characterization, particularly given that Fiori et al. (2017) demonstrated PMv facilitation at 80 ms and long-latency IHI at 150 ms. However, we prioritized specific ISIs to compare well-established mechanisms (ie short- and long-IHI) alongside novel non-callosal IHI (0 ms ISI) motivated by non-human primate work (Quessy et al. 2016; Côté et al. 2017). We did not vary conditioning stimulus current direction in the current study; although prior work suggests limited effects on IHI elicited from motor and premotor regions (Mochizuki et al. 2004; Ni et al. 2009), potential region-specific influences remain incompletely understood. Fourth, we assessed conditioning of the left (dominant-hemisphere) M1 by right-hemisphere sites only (ie a single interhemispheric direction). Because IHI can be asymmetric with respect to hemispheric dominance and direction, our findings do not generalize to left-to-right premotor influences and should be interpreted accordingly. Future work should assess both directions and any PMd/PMv asymmetries. Finally, while our non-callosal IHI findings at 0 ms ISI are novel, we can only speculate about convergence sites of subcortical structures (reticular formation, brainstem), the spinal cord, or both. Since transcallosal conduction in humans requires at least 4 to 5 ms to modulate contralateral M1 (Hanajima et al. 2001; Ni et al. 2020), we are relatively confident the 0 ms ISI inhibition was not transcallosally mediated. Nevertheless, while the 0 ms ISI findings are robust, we can only speculate about the underlying mechanisms. The present data do not allow us to distinguish between convergence at brainstem, spinal, or other subcortical levels, nor to exclude potential non-specific effects of simultaneous stimulation. These possibilities should be directly tested in future studies.

## Conclusion

This study provides a comprehensive characterization of the differential modulation of contralateral M1 excitability by PMd and PMv in humans. We demonstrate that an iSP can be elicited from PMd and PMv, revealing evidence of differential transcallosal inhibitory connectivity with contralateral M1. Additionally, our dual-site TMS approach mapped short-, long-, and non-callosal interhemispheric inhibitory mechanisms as elicited from PMd, PMv, and M1. Notably, the association between iSP duration and long-IHI magnitude from both PMd and PMv suggests these measures reflect partly overlapping transcallosal mechanisms, bridging our single-pulse and dual-site findings. Our results provide a framework for further investigation into the mechanisms by which distinct premotor cortical regions, like PMd and PMv, shape M1 output. Finally, our findings of differential connectivity patterns may be important when considering neuromodulation strategies targeting premotor regions in stroke rehabilitation.

## Supplementary Material

Supplementary_Materials_Table_1_bhag163

## Data Availability

All main data presented in this manuscript are included within the figures. The datasets produced and analyzed in this study are available from the corresponding author upon reasonable request.

## References

[ref1] Bäumer T et al. 2006. Magnetic stimulation of human premotor or motor cortex produces interhemispheric facilitation through distinct pathways. J Physiol. 572:857–868. 10.1113/jphysiol.2006.104901. PMC1780006 16497712

[ref2] Bäumer T et al. 2009. Inhibitory and facilitatory connectivity from ventral premotor to primary motor cortex in healthy humans at rest - a bifocal TMS study. Clin Neurophysiol. 120:1724–1731. 10.1016/j.clinph.2009.07.035. 19683960

[ref3] Bestmann S et al. 2010. The role of contralesional dorsal premotor cortex after stroke as studied with concurrent TMS-fMRI. J Neurosci. 30:11926–11937. 10.1523/JNEUROSCI.5642-09.2010. PMC3044467 20826657

[ref4] Borich MR, Neva JL, Boyd LA. 2015. Evaluation of differences in brain neurophysiology and morphometry associated with hand function in individuals with chronic stroke. Restor Neurol Neurosci. 33:31–42. 10.3233/RNN-140425. 25374346

[ref5] Boroojerdi B, Diefenbach K, Ferbert A. 1996. Transcallosal inhibition in cortical and subcortical cerebral vascular lesions. J Neurol Sci. 144:160–170. 10.1016/S0022-510X(96)00222-5. 8994119

[ref6] Borra E, Belmalih A, Gerbella M, Rozzi S, Luppino G. 2010. Projections of the hand field of the macaque ventral premotor area F5 to the brainstem and spinal cord. J Comp Neurol. 518:2570–2591. 10.1002/cne.22353. 20503428

[ref7] Boussaoud D, Tanné-Gariépy J, Wannier T, Rouiller EM. 2005. Callosal connections of dorsal versus ventral premotor areas in the macaque monkey: a multiple retrograde tracing study. BMC Neurosci. 6:67. 10.1186/1471-2202-6-67 PMC1314896 16309550

[ref8] Chen R, Yung D, Li J-Y. 2003. Organization of Ipsilateral Excitatory and Inhibitory Pathways in the human motor cortex. J Neurophysiol. 89:1256–1264. 10.1152/jn.00950.2002. 12611955

[ref9] Chen R et al. 2008. The clinical diagnostic utility of transcranial magnetic stimulation: report of an IFCN committee. Clin Neurophysiol. 119:504–532. 10.1016/j.clinph.2007.10.014. 18063409

[ref10] Chouinard PA, Paus T. 2006. The primary motor and premotor areas of the human cerebral cortex. Neuroscientist. 12:143–152. 10.1177/1073858405284255. 16514011

[ref11] Cohen J. 2013. Statistical power analysis for the Behavioral sciences. 2nd ed. Routledge.

[ref12] Côté SL, Hamadjida A, Quessy S, Dancause N. 2017. Contrasting modulatory effects from the dorsal and ventral premotor cortex on primary motor cortex outputs. J Neurosci. 37:5960–5973. 10.1523/JNEUROSCI.0462-17.2017. PMC6596508 28536271

[ref13] Dancause N . 2006. Neurophysiological and anatomical plasticity in the adult sensorimotor cortex. Rev Neurosci. 17:561–580. 10.1515/REVNEURO.2006.17.6.561. 17283605

[ref14] Dancause N, Barbay S, Frost SB, Mahnken JD, Nudo RJ. 2007. Interhemispheric connections of the ventral premotor cortex in a new world primate. J Comp Neurol. 505:701–715. 10.1002/cne.21531. PMC3266721 17948893

[ref15] Dancause N et al. 2005. Extensive cortical rewiring after brain injury. J Neurosci. 25:10167–10179. 10.1523/JNEUROSCI.3256-05.2005. PMC6725801 16267224

[ref16] Daskalakis ZJ, Christensen BK, Fitzgerald PB, Roshan L, Chen R. 2002. The mechanisms of interhemispheric inhibition in the human motor cortex. J Physiol. 543:317–326. 10.1113/jphysiol.2002.017673. PMC2290496 12181302

[ref17] Davare M, Lemon R, Olivier E. 2008. Selective modulation of interactions between ventral premotor cortex and primary motor cortex during precision grasping in humans. J Physiol-London. 586:2735–2742. 10.1113/jphysiol.2008.152603. PMC2536583 18403420

[ref18] Davare M, Montague K, Olivier E, Rothwell JC, Lemon RN. 2009. Ventral premotor to primary motor cortical interactions during object-driven grasp in humans. Cortex. 45:1050–1057. 10.1016/j.cortex.2009.02.011 PMC2730595 19345344

[ref19] Davidson T, Tremblay F. 2013. Age and hemispheric differences in transcallosal inhibition between motor cortices: an ipsilateral silent period study. BMC Neurosci. 14:62. 10.1186/1471-2202-14-62 PMC3695846 23800346

[ref20] Dea M, Hamadjida A, Elgbeili G, Quessy S, Dancause N. 2016. Different patterns of cortical inputs to subregions of the primary motor cortex hand representation in *Cebus apella*. Cereb Cortex. 26:1747–1761. 10.1093/cercor/bhv324. PMC4785954 26966266

[ref21] Denyer R, Greenhouse I, Boyd LA. 2023. PMd and action preparation: bridging insights between TMS and single neuron research. Trends Cogn Sci. 27:759–772. 10.1016/j.tics.2023.05.001. 37244800

[ref22] Eickhoff SB et al. 2005. A new SPM toolbox for combining probabilistic cytoarchitectonic maps and functional imaging data. NeuroImage. 25:1325–1335. 10.1016/j.neuroimage.2004.12.034 15850749

[ref23] Ferbert A et al. 1992. Interhemispheric inhibition of the human motor cortex. J Physiol. 453:525–546. 10.1113/jphysiol.1992.sp019243. PMC1175572 1464843

[ref24] Fiori F et al. 2017. Long-latency interhemispheric interactions between motor-related areas and the primary motor cortex: a dual site TMS study. Sci Rep. 7:10. 10.1038/s41598-017-13708-2 PMC5668244 29097700

[ref25] Fisher KM, Zaaimi B, Baker SN. 2012. Reticular formation responses to magnetic brain stimulation of primary motor cortex. J Physiol. 590:4045–4060. 10.1113/jphysiol.2011.226209. PMC3464356 22674723

[ref26] Fling BW, Seidler RD. 2012. Task-dependent effects of interhemispheric inhibition on motor control. Behav Brain Res. 226:211–217. 10.1016/j.bbr.2011.09.018. PMC3208314 21944939

[ref27] Fregosi M, Contestabile A, Hamadjida A, Rouiller EM. 2017. Corticobulbar projections from distinct motor cortical areas to the reticular formation in macaque monkeys. Eur J Neurosci. 45:1379–1395. 10.1111/ejn.13576. 28394483

[ref28] Frost SB, Barbay S, Friel KM, Plautz EJ, Nudo RJ. 2003. Reorganization of remote cortical regions after ischemic brain injury: a potential substrate for stroke recovery. J Neurophysiol. 89:3205–3214. 10.1152/jn.01143.2002. 12783955

[ref29] Gamst G, Meyers LS, Guarino AJ. 2008. Analysis of variance designs: a conceptual and computational approach with SPSS and SAS. Cambridge University Press, and Place, New York, NY, Year, p 2008.

[ref30] Ghosh S et al. 2013. Short- and long-latency interhemispheric inhibitions are additive in human motor cortex. J Neurophysiol. 109:2955–2962. 10.1152/jn.00960.2012. 23536711

[ref31] Grefkes C, Eickhoff SB, Nowak DA, Dafotakis M, Fink GR. 2008. Dynamic intra-and interhemispheric interactions during unilateral and bilateral hand movements assessed with fMRI and DCM. NeuroImage. 41:1382–1394. 10.1016/j.neuroimage.2008.03.048 18486490

[ref32] Groppa S et al. 2012. The human dorsal premotor cortex facilitates the excitability of ipsilateral primary motor cortex via a short latency cortico-cortical route. Hum Brain Mapp. 33:419–430. 10.1002/hbm.21221. PMC6869901 21391274

[ref33] Hanajima R et al. 2001. Interhemispheric facilitation of the hand motor area in humans. J Physiol. 531:849–859. 10.1111/j.1469-7793.2001.0849h.x. PMC2278503 11251064

[ref34] Hoshi E, Tanji J. 2004. Functional specialization in dorsal and ventral premotor areas. In: Editors: S. Mori DGS, M. Wiesendanger, editor. Progress in Brain Research Elsevier p 507–511, DOI: 10.1021/la036149g. 14653192

[ref35] Hoshi E, Tanji J. 2007. Distinctions between dorsal and ventral premotor areas: anatomical connectivity and functional properties. Curr Opin Neurobiol. 17:234–242. 10.1016/j.conb.2007.02.003. 17317152

[ref36] Hui J et al. 2020. Pharmacological mechanisms of interhemispheric signal propagation: a TMS-EEG study. Neuropsychopharmacology. 45:932–939. 10.1038/s41386-019-0468-7 PMC7162860 31357206

[ref37] Hupfeld KE, Swanson CW, Fling BW, Seidler RD. 2020. TMS-induced silent periods: a review of methods and call for consistency. J Neurosci Methods. 346:108950. 10.1016/j.jneumeth.2020.108950. PMC8276277 32971133

[ref38] Irlbacher K, Brocke J, Jv M, Brandt SA. 2007. Effects of GABAA and GABAB agonists on interhemispheric inhibition in man. Clin Neurophysiol. 118:308–316. 10.1016/j.clinph.2006.09.023. 17174150

[ref39] Jung P, Ziemann U. 2006. Differences of the ipsilateral silent period in small hand muscles. Muscle Nerve. 34:431–436. 10.1002/mus.20604. 16810689

[ref40] Kably B, Drew T. 1998. Corticoreticular pathways in the cat. I. Projection patterns and Collaterization. J Neurophysiol. 80:389–405. 10.1152/jn.1998.80.1.389. 9658059

[ref41] Keizer K, Kuypers HGJM. 1989. Distribution of corticospinal neurons with collaterals to the lower brain stem reticular formation in monkey (Macaca fascicularis). Exp Brain Res. 74(2):311–318. 10.1007/BF00248864. 2924851

[ref42] Koch G et al. 2006. Time course of functional connectivity between dorsal premotor and contralateral motor cortex during movement selection. J Neurosci. 26:7452–7459. 10.1523/JNEUROSCI.1158-06.2006. PMC6674199 16837593

[ref43] Kujirai T et al. 1993. Corticocortical inhibition in human motor cortex. J Physiol. 471:501–519. 10.1113/jphysiol.1993.sp019912. PMC1143973 8120818

[ref44] Lakens D . 2022. Sample size justification. Collabra. Psychology. 8:33267.

[ref45] Mang CS et al. 2015. Diffusion imaging and transcranial magnetic stimulation assessment of transcallosal pathways in chronic stroke. Clin Neurophysiol. 126:1959–1971. 10.1016/j.clinph.2014.12.018. 25631612

[ref46] Marconi B, Genovesio A, Giannetti S, Molinari M, Caminiti R. 2003. Callosal connections of dorso-lateral premotor cortex. Eur J Neurosci. 18:775–788. 10.1046/j.1460-9568.2003.02807.x. 12925004

[ref47] Merrell LH et al. 2024. Myths and methodologies: standardisation in human physiology research—should we control the controllables? Exp Physiol. 109:1099–1108. 10.1113/EP091557. PMC11215472 38763158

[ref48] Meyer BU, Röricht S, Von Einsiedel HG, Kruggel F, Weindl A. 1995. Inhibitory and excitatory interhemispheric transfers between motor cortical areas in normal humans and patients with abnormalities of the corpus callosum. Brain. 118:429–440. 10.1093/brain/118.2.429 7735884

[ref49] Mochizuki H, Huang YZ, Rothwell JC. 2004. Interhemispheric interaction between human dorsal premotor and contralateral primary motor cortex. J Physiol. 561:331–338. 10.1113/jphysiol.2004.072843. PMC1665328 15459244

[ref50] Morecraft RJ et al. 2019. Terminal organization of the corticospinal projection from the lateral premotor cortex to the cervical enlargement (C5–T1) in rhesus monkey. J Comp Neurol. 527:2761–2789. 10.1002/cne.24706. PMC6721988 31032921

[ref51] Neva JL, Hayward KS, Boyd LA. 2020. Therapeutic effects of repetitive transcranial magnetic stimulation (rTMS) in stroke: Moving toward an individualized approach. In: The Wiley Encyclopedia of Health Psychology Sweeny K, Robbins ML, Cohen LM (eds), 1st edn. Wiley, pp 169–179.

[ref52] Neva JL et al. 2016. Multiple measures of corticospinal excitability are associated with clinical features of multiple sclerosis. Behav Brain Res. 297:187–195. 10.1016/j.bbr.2015.10.015. PMC4904787 26467603

[ref53] Neva JL, Vesia M, Singh AM, Staines WR. 2015. Bilateral primary motor cortex circuitry is modulated due to theta burst stimulation to left dorsal premotor cortex and bimanual training. Brain Res. 1618:61–74. 10.1016/j.brainres.2015.05.028. 26032743

[ref54] Ni Z et al. 2009. Two phases of interhemispheric inhibition between motor related cortical areas and the primary motor cortex in human. Cereb Cortex. 19:1654–1665. 10.1093/cercor/bhn201. 19015374

[ref55] Ni Z et al. 2020. Measuring latency distribution of transcallosal fibers using transcranial magnetic stimulation. Brain Stimul. 13:1453–1460. 10.1016/j.brs.2020.08.004. PMC7417270 32791313

[ref56] O'Shea J, Sebastian C, Boorman ED, Johansen-Berg H, Rushworth MFS. 2007. Functional specificity of human premotor-motor cortical interactions during action selection. Eur J Neurosci. 26:2085–2095. 10.1111/j.1460-9568.2007.05795.x. 17868374

[ref57] Oldfield RC . 1971. The assessment and analysis of handedness: the Edinburgh inventory. Neuropsychologia. 9:97–113. 10.1016/0028-3932(71)90067-4 5146491

[ref58] Opitz A, Windhoff M, Heidemann RM, Turner R, Thielscher A. 2011. How the brain tissue shapes the electric field induced by transcranial magnetic stimulation. NeuroImage. 58:849–859. 10.1016/j.neuroimage.2011.06.069 21749927

[ref59] Paul T et al. 2023. Interhemispheric structural connectivity underlies motor recovery after stroke. Ann Neurol. 94:785–797. 10.1002/ana.26737. 37402647

[ref60] Picard N, Strick PL. 2001. Imaging the premotor areas. Curr Opin Neurobiol. 11:663–672. 10.1016/S0959-4388(01)00266-5. 11741015

[ref61] Quessy S, Côté SL, Hamadjida A, Deffeyes J, Dancause N. 2016. Modulatory effects of the Ipsi and contralateral ventral premotor cortex (PMv) on the primary motor cortex (M1) outputs to intrinsic hand and forearm muscles in *Cebus apella*. Cereb Cortex. 26:3905–3920. 10.1093/cercor/bhw186. PMC5028004 27473318

[ref62] Rizzolatti G, Luppino G. 2001. The cortical motor system. Neuron. 31:889–901. 10.1016/s0896-6273(01)00423-8 11580891

[ref63] Rossini PM et al. 1994. Non-invasive electrical and magnetic stimulation of the brain, spinal cord and roots: basic principles and procedures for routine clinical application. Report of an IFCN committee. Electroencephalogr Clin Neurophysiol. 91:79–92. 10.1016/0013-4694(94)90029-9. 7519144

[ref64] Ruddy KL, Leemans A, Carson RG. 2017. Transcallosal connectivity of the human cortical motor network. Brain Struct Funct. 222:1243–1252. 10.1007/s00429-016-1274-1. PMC5368198 27469272

[ref65] Schulz R et al. 2015. White matter integrity of premotor–motor connections is associated with motor output in chronic stroke patients. Neuroimage Clin. 7:82–86. 10.1016/j.nicl.2014.11.006. PMC4299958 25610769

[ref66] Tamm AS, Lagerquist O, Ley AL, Collins DF. 2009. Chronotype influences diurnal variations in the excitability of the human motor cortex and the ability to generate torque during a maximum voluntary contraction. J Biol Rhythm. 24:211–224. 10.1177/0748730409334135. 19465698

[ref67] Thielscher A, Kammer T. 2004. Electric field properties of two commercial figure-8 coils in TMS: calculation of focality and efficiency. Clin Neurophysiol. 115:1697–1708. 10.1016/j.clinph.2004.02.019. 15203072

[ref68] Udupa K, Ni Z, Gunraj C, Chen R. 2010. Effect of long interval interhemispheric inhibition on intracortical inhibitory and facilitatory circuits. J Physiol Lond. 588:2633–2641. 10.1113/jphysiol.2010.189548. PMC2916993 20519316

[ref69] Van Malderen S, Hehl M, Verstraelen S, Swinnen SP, Cuypers K. 2023. Dual-site TMS as a tool to probe effective interactions within the motor network: a review. Rev Neurosci. 34:129–221. 10.1515/revneuro-2022-0020. 36065080

[ref70] Wise SP . 2006. The ventral premotor cortex, corticospinal region C, and the origin of primates. Cortex. 42:521–524. 10.1016/s0010-9452(08)70391-5 16881263

[ref71] Yarkoni T, Poldrack RA, Nichols TE, Van Essen DC, Wager TD. 2011. Large-scale automated synthesis of human functional neuroimaging data. Nat Methods. 8:665–670. 10.1038/nmeth.1635. PMC3146590 21706013

[ref72] Zarei M et al. 2006. Functional anatomy of interhemispheric cortical connections in the human brain. J Anat. 209:311–320. 10.1111/j.1469-7580.2006.00615.x. PMC2100336 16928200

