# Supplementary material for: Premotor cortices differentially influence contralateral motor cortex
Source: Cereb Cortex. 2026 Oct 12;36(10):bhag163. doi: 10.1093/cercor/bhag163 (PMC13657042; doi:10.1093/cercor/bhag163)
Supplement: Supplementary file 1 [file supplementary_materials_table_1_bhag163.docx]

**SUPPLEMENTARY MATERIALS**

**Title:** Premotor cortices differentially influence contralateral motor cortex

**Running title:** PMd and PMv differentially influence contralateral M1

**Elnaz Allahverdloo^2,3,4^, Larissa K. Chiu^2,^ Amanda O’Farrell^1,2^, Nesrine Harroum^1,2^, Numa Dancause^3,4,5^, and Jason L. Neva^1,2,4 *^**

**Affiliations :**

^1^École de kinésiologie et des sciences de l'activité physique, Université de Montréal, Montréal, QC, Canada

^2^Centre de recherche de l’institut universitaire de gériatrie de Montréal, Montréal, QC, Canada ;

^3^Département de neurosciences, Université de Montréal, Montréal, QC, Canada ;

^4^Centre Interdisciplinaire de Recherche sur le Cerveau et l’Apprentissage (CIRCA), Université de Montréal, Montréal, QC, Canada

^5^Institut Courtois d’innovation biomédicale (CI^2^B), Université de Montréal, Montréal, QC, Canada

**^*^Corresponding Author:**

Jason L. Neva, PhD

École de kinésiologie et des sciences de l'activité physique

2100, boul. Édouard-Montpetit

C.P. 6128, succursale Centre-ville

Montréal (Québec) H3C 3J7

Canada

Email: jason.neva@umontreal.ca

Twitter / X handle: @jasonlneva

| TS | M1 | PMd | PMv |
| --- | --- | --- | --- |
| TS Intensity (% MSO) | 55.7 (11.1) | 55.26 (9.9) | 55.5 (10.3) |
| TS Amplitude (mV) | 1.39 (0.39) | 1.35 (0.47) | 1.40 (0.72) |

**Supplementary Table 1: Test Stimulus data**

Data shown is the peak-to-peak amplitude of the motor evoked potential expressed in mV and intensity of test stimulus expressed in % MSO reported as mean (SD). Abbreviations: **M1:** primary motor cortex, **PMd:** dorsal premotor cortex, **PMv:** ventral premotor cortex
